# Insights into the Genomic Architecture and Improvement of the Capabilities of *Acinetobacter calcoaceticus* for the Biodegradation of Petroleum Hydrocarbons

**DOI:** 10.3390/microorganisms13081953

**Published:** 2025-08-21

**Authors:** Yaning Zeng, Mutian Wang, Xiaoyu Chang, Leilei Wang, Xiaowen Fu, Yujie Huang, Fanyong Song, Lei Ji, Jianing Wang

**Affiliations:** 1 Shandong Provincial Key Laboratory of Applied Microbiology, Ecology Institute, Qilu University of Technology (Shandong Academy of Sciences), Jinan 250103, China; 2 EnviroCORE, Dargan Research Centre, South East Technological University, Carlow Campus, R93 V960 Carlow, Ireland; 3 Key Laboratory of Pollution Processes and Environmental Criteria (Ministry of Education), College of Environmental Science and Engineering, Nankai University, Tianjin 300350, China

**Keywords:** petroleum-hydrocarbons biodegradation, genome analysis, metabolic pathways, *Acinetobacter calcoaceticus*, prokaryotic expression, subterminal oxidation

## Abstract

Petroleum-contaminated terrestrial ecosystems require effective bioremediation strategies. In this study, genomic analysis revealed key biodegradation genes on the *Acinetobacter calcoaceticus* 21# chromosome: alkane hydroxylases (*alkB*, *almA*, *LadA*) and aromatic ortho-cleavage pathway genes (*catABC*). Phylogenetic and multiple sequence alignment analyses of the *almA* gene in strain 21# revealed the presence of signature motifs characteristic of Baeyer–Villiger monooxygenase. Functional annotation analysis demonstrated stronger phylogenetic affinity of this protein to previously characterized BVMOs than to hydroxylases. Therefore, it is suggested that the AlmA protein in 21# exhibits BVMO activity and participates in the subterminal oxidation pathway of alkane degradation. Wild-type 21# degraded both n-Octacosane (24.47%) and pyrene (34.03%). Engineered 21#-A3 showed significantly enhanced n-Octacosane degradation (28.68%). To validate AlmA function and assess impacts of exogenous gene integration, we expressed the *almA* gene from *A. vivianii* KJ-1 via pET-28a(+)-*av-almA-BH* vector. Enzymatic assays demonstrated no activity toward long-chain alkanes but high activity for 2-decanone (0.39 U/mg) and 2-dodecanone (0.37 U/mg). Metabolite analysis confirmed recombinant AlmA functions through subterminal oxidation. This study establishes a foundational framework for advancing the optimization of petroleum-degrading bacteria. To engineer more efficient hydrocarbon-degrading strains, future research should integrate meta-cleavage pathways to expand their substrate utilization range for polycyclic aromatic hydrocarbons.

## 1. Introduction

Petroleum remains a critical energy resource in modern industrial systems; however, soil contamination caused by leakage incidents during its extraction, transportation, and storage has intensified, leading to hydrocarbon diffusion in terrestrial ecosystems [1,2,3]. Although indigenous microorganisms possess inherent remediation capabilities [4,5], the natural attenuation processes in oilfield-contaminated soils require extended durations to achieve safety thresholds for hydrocarbon concentrations, posing persistent ecological risks [6].

To date, over 200 species across 90 genera of soil-derived microorganisms with hydrocarbon-degrading capabilities have been documented, including but not limited to *Pseudomonas* [7,8], *Bacillus* [8,9], *Rhodococcus* [10,11], *Nocardia*, *Acinetobacter* and *Microbacterium*. Notably, *Acinetobacte*, a representative alkane-degrading genus, constitutes one of the primary microbial degraders of environmental alkanes. *A. venetianus* 2AW degrades medium-chain alkanes [12], while *A. radioresistens* APH1 exhibits phenol removal and plant-microbe synergy [13]. In previous studies, the assessment of microbial degradation capabilities has predominantly focused on medium-chain alkanes or simple aromatic hydrocarbons with fewer benzene rings. Research on long-chain alkanes and highly condensed polycyclic aromatic hydrocarbons (PAHs) remains limited, and microbial strains exhibiting efficient co-degradation of both recalcitrant substrates are particularly scarce.

During the initial phase of this research, a wild-type, high-efficiency strain, *A. calcoaceticus* 21#, isolated from petroleum-contaminated soil in the Shengli Oilfield demonstrated significant petroleum degradation activity. Concurrently, *A. vivianii* KJ-1 exhibited robust alkane degradation capabilities [14]. Building upon these preliminary findings, we conducted genomic mining of *A. calcoaceticus* 21# to identify genes and metabolic pathways associated with hydrocarbon degradation. Subsequently, we performed a comparative analysis of hydrocarbon degradation between the wild-type strain and the genetically engineered strain. This was performed to investigate whether the introduction of the foreign gene confers novel or enhanced functional characteristics and to assess its impact on the original degradation capability. This approach eliminates confounding factors arising from the significant genomic and physiological differences between their native hosts, thereby providing a more direct and clear demonstration of the functional role of this gene segment. To elucidate the mechanistic basis of enhanced degradation, the *almA* gene from *A. vivianii* KJ-1 was heterologously expressed in a prokaryotic host, and its functional role was further validated. This study aims to lay the groundwork for developing high-performance petroleum-degrading consortia through targeted genetic engineering.

## 2. Materials and Methods

### 2.1. Chemicals and Medium

2-Decanone (≥98%), 2-Dodecanone (≥98%), n-Octacosane (C_28_H_58_, 98%), Pyrene (Pyr, 97%), flavin adenine dinucleotide (FAD, ≥95%), and β-nicotinamide adenine dinucleotide phosphate (NADPH, ≥97%) were procured from Macklin Inc. (Shanghai, China). All other chemicals were of analytical grade.

LB Medium: 10 g tryptone, 10 g NaCl, and 5 g yeast extract were dissolved in 1 L of deionized water.

Mineral Salt Medium (MSM): 0.5 g K_2_HPO_4_, 2.0 g Na_2_SO_4_, 1.0 g NH_4_Cl, 0.02 g MgSO_4_·7H_2_O, 0.07 g CaCl_2_, and 1.0 mL of trace salt solution. The trace salt solution is defined as containing 0.03 g FeCl_3_, 0.0005 g CuSO_4_, 0.0005 g MnSO_4_·H_2_O, and 0.01 g ZnSO_4_·7H_2_O per liter. The pH was adjusted to 7.0–7.2 using 1 M HCl or NaOH.

### 2.2. Whole-Genome Sequencing Analysis of A. calcoaceticus 21#

*A. calcoaceticus* 21# (Table 1) was streaked onto agar plates. A single colony was inoculated into LB broth and cultured to the logarithmic growth phase under shaking conditions. Cells were harvested by centrifugation at 7000 rpm for 5 min (4 °C), washed three times with sterile phosphate-buffered saline, and flash-frozen in liquid nitrogen for ≥15 min prior to storage at −80 °C.

Libraries with varying insert sizes were constructed and sequenced on both the Illumina NovaSeq platform (PE 2 × 150 bp) and the PacBio Sequel platform. Primary assembly of PacBio long reads was conducted using Unicycler v0.5.0 and Flye v2.9, generating contig sequences. Short Illumina reads were assembled using SPAdes [15] and A5-miseq [16] to construct scaffolds and contigs. All assembled results were integrated to generate a complete sequence. Illumina short reads were subsequently employed for error correction via Pilon v1.24 to refine the assembly. Functional annotation of protein-coding genes in 21# was conducted to elucidate molecular-level functional traits of the strain using GeneMarkS v4.32 [17] for prediction. Functional prediction of these genes was achieved through comparative analysis against functionally character-ized protein sequences in publicly available databases, including the KEGG (Kyoto Encyclopedia of Genes and Genomes) database [18].

### 2.3. Degradation Efficiency Assay

Long-chain alkane degradation assay. *A. calcoaceticus* 21# was inoculated into LB medium and cultured at 30 °C with shaking (150 rpm). The bacterial cells were collected at 4 °C, washed twice with sterile phosphate-buffered saline (PBS, pH 7.4), and resuspended to make the final optical density (OD_600_) reach 0.6. The bacterial suspension (2% inoculum) was transferred into MSM medium containing 1 g/L n-Octacosane and cultured for 7 days. Samples were collected to quantify residual n-Octacosane. Uninoculated MSM containing n-Octacosane served as the abiotic control. All experiments were performed in triplicate to ensure statistical reliability. Liquid–liquid extraction was performed using a 2:1 (*V*/*V*) ratio of culture supernatant to *n*-hexane. The organic phase was concentrated via rotary evaporation, reconstituted in *n*-hexane. Processed samples were stored in GC vials for analysis. The concentrations of n-Octacosane were determined using an Agilent 7890B gas chromatograph (Agilent Technologies, Santa Clara, CA, USA) equipped with a flame ionization detector (GC-FID) and an HP-5 capillary column (30 m × 0.32 mm × 0.25 μm) (Agilent Technologies, Santa Clara, CA, USA). The GC-FID operational parameters were set as follows: nitrogen carrier gas flow at 1.5 mL/min and hydrogen and air flows maintained at 50 mL/min and 300 mL/min, respectively. Splitless injection mode was employed with a 1 μL injection volume. The injector port and detector temperatures were held at 300 °C and 325 °C, respectively. The column oven temperature program comprised an initial isothermal phase at 50 °C (2 min), followed by a temperature ramp to 230 °C at 40 °C /min and a secondary ramp to 320 °C at 20 °C /min with a final 3 min hold.

Determination of Polycyclic Aromatic Hydrocarbon (PAH) degradation assay. The bacterial suspension (2% *V*/*V* inoculum) was transferred into MSM supplemented with 50 mg/L Pyr. The remaining procedures were identical to the experimental protocol for n-Octacosane (dichloromethane extraction and methanol reconstitution). PAH concentrations were determined using high-performance liquid chromatography (HPLC; Agilent 1260 Infinity II) under conditions adapted from the Chinese National Environmental Protection Standard HJ 478-2009 [19]. The HPLC operating parameters are as follows: The mobile phase comprised methanol/water (9:1, *v*/*v*), delivered at a flow rate of 1.0 mL/min under isocratic elution. The column temperature was maintained at 25 °C, with a system pressure of 600 Pa. Detection wavelengths were set at 240 nm for Pyr. An injection volume of 5 µL was utilized, and the total run time was 10 min.

### 2.4. Construction of Expression Vectors

The *A. vivianii* KJ-1 genome was used as a template for PCR amplification using primers F-B-*av-almA*-*BH* (5′-3′:CGGGATCCATGGAAAAGCAAGTTGACGTATT) and R-H-*av-almA*-*BH* (5′-3′:CCCAAGCTTCGATACCAGTTTTGGTTTACGA), respectively. The *av-almA* with BamH I, Hind III restriction site, were ligated in pLB vector and then transferred into *E.coli* DH5α competent cells by heat-stimulated transformation. Transformants were screened using LB plates containing 100 μg/mL ampicillin. The pLB-*almA-BH* and pET28a(+) were digested using BamH I and Hind III restriction endonuclease, respectively. The digest products were then recovered and ligated using T4 DNA ligase. As a result, the recombinant expression vector pET28a(+)-*almA-BH* was constructed. Subsequently, the vector was transformed into *E. coli* BL21(DE3) competent cells by heat-stimulated transformation and the transformants were screened using LB plates containing 50 μg/mL kanamycin.

### 2.5. Expression of av-almA Gene in E. coli BL21(DE3) and Purification of Recombinant Protein av-almA

*E. coli* BL21(DE3) cells that express *av-almA* were cultured in 1 L LB media supplemented with 50 μg/mL kanamycin at 37 °C with shaking at 150 rpm to approximately OD_600_ = 0.6. The expression of the *av-almA* gene was induced by the addition of 0.2 mM Isopropyl β-D-thiogalactoside (IPTG), followed by incubation at 37 °C for 4 h. The cells were collected through centrifugation at 7000 rpm (Eppendorf Centrifuge 5804R, Hamburg, Germany) at 4 °C for 5 min and then washed and resuspended in 50 mM Tris-HCl buffer (pH 7.4). the buffer was added to the cell in the ratio of 10:1 (*v*/*w*). After the addition of phenylmethylsulphonyl fluoride and lysozyme (final concentrations of 1 mM and 1 mg/mL, respectively), the cell suspension was incubated at 4 °C for 20 min and then sonicated at 195 W for 6 min with sonication time 3 s and quench time 5 s. Finally, the supernatant was collected by centrifugation at 10,000 rpm at 4 °C as the av-almA-containing enzyme solution. Protein purification was performed using a Ni^2+^ column according to the procedure recommended by the manufacturer (Beyotime Biotechnology, Shanghai, China). The nickel-affinity resin was pre-equilibrated by centrifugation (2–3 cycles). Subsequently, 4 mL of bacterial lysate supernatant was added and the mixture was incubated at 4 °C with gentle shaking (100 rpm) for 60 min to facilitate protein binding. The resin-lysate mixture was then transferred to a chromatography column, and a 20 μL aliquot of the initial gravity flow-through was collected for quality control. The resin was washed rigorously with 0.5 mL of non-denaturing lysis buffer per wash for a total of four washes, with the wash fraction collected each time to thoroughly remove contaminants. Finally, the target protein was eluted by applying 0.5 mL of non-denaturing elution buffer in six sequential aliquots (0.5 mL × 6); each elution fraction was collected separately, yielding high-purity His-tagged protein samples. Aliquots (20 μL) of the purified samples were transferred to clean microcentrifuge tubes, mixed with an equal volume of 2× SDS-PAGE loading buffer, and incubated at 100 °C in a metal bath for 10 min. Samples were then centrifuged at 13,000 rpm for 5 min, and 5 μL of the resulting supernatant was loaded for SDS-PAGE gel electrophoresis.

### 2.6. Activity Assay of av-almA Protein

Enzyme activity was calculated by the difference in the change in absorbance values of the coenzyme NADPH at the characteristic absorption peak at 340 nm. One unit of enzyme activity is defined as the amount of enzyme required to consume 1 μmol of NADPH per minute. The reaction mixture consisted of 50 mM Tris-HCl (pH 7.4), 1 mM 2-decanone or 2-dodecanone, 0.5 mM NADPH, 0.2 mM FAD, and a suitable amount of purified enzyme. The reaction was carried out at 25 °C and the total volume of the reaction was 1 mL. All experiments were performed in triplicate. The formula for calculating enzyme activity is as follows:

*U* = ∆340 × *V* × 10^3^/6220 × *L* (1)

where ∆340 denotes the difference in the change in absorbance of coenzyme NADPH at 340 nm per minute, 6220 is the molar absorption coefficient (L/mol·cm), and *L* is the optical distance.

### 2.7. Metabolite Profiling of Recombinant AlmA Protein

The reaction mixture (5 mL) consisted of 50 mM Tris-HCl (pH 7.4), 1 mM n-Octacosane or aliphatic ketones, 1 mM NADPH, 0.2 mM FAD, and 1 mg purified enzyme. The above mixtures were allowed to react for 1 h at 30 °C and then extracted with n-Hexane. The resulting products of the enzymatic reactions were analyzed using a GC–MS system (Thermo, Waltham, MA, USA) consisting of Trace 1300-GC equipped with an HP-5MS separation column (30 m × 0.25 mm × 0.25 μm) and ISQ7000-MS detector.

## 3. Results and Discussion

### 3.1. Genomic Insights into A. calcoaceticus 21#

The complete genome of *A. calcoaceticus* 21# comprises a single circular chromosome (3,839,750 bp; GC content: 38.67%) with no detectable plasmids. A total of 3829 protein-coding genes were identified, accounting for 87.03% of the genome length (Figure S1). KEGG analysis identified 1434 genes (37.45%) involved in metabolic pathways, with 148 genes annotated to xenobiotic biodegradation (Figure S2). Key pathways include benzoate degradation (ko00362), fatty acid metabolism (ko00071), chloroalkane/alkene degradation (ko00625), naphthalene degradation (ko00626), and toluene/xylene degradation (ko00622). These pathways are directly implicated in the breakdown of petroleum constituents. Our analysis revealed that *A. calcoaceticus* 21# exhibited a rich repertoire of metabolic pathway-related genes in terms of both quantity and functional variety, confirming its molecular-level hydrocarbon degradation capacity and demonstrating its potential as a bioremediation agent for petroleum pollutants.

#### 3.1.1. Alkane Degradation-Related Genes

**Figure 1:**
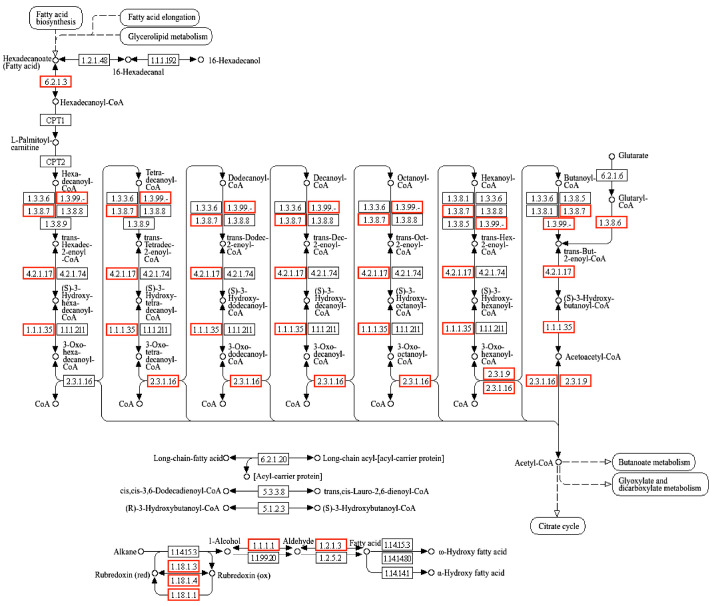
Metabolic pathway of putative strain *A. calcoaceticus* 21# to medium chain alkanes. The red box indicates that the genome of *A. calcoaceticus* 21# contains genes.

Mid-Chain Alkane Degradation Gene. Genes associated with mid-chain alkane degradation in 21# are listed in Table 2. Two key genes, *alkB*1_1 and *alkB*1_2, encoding membrane-bound non-heme di-iron alkane hydroxylases (AlkB), were identified. These enzymes catalyze the terminal oxidation of alkanes, a process dependent on iron cofactors. Over 60 homologs of AlkB proteins have been identified and are widely conserved across diverse bacterial taxa [20]. AlkB-mediated hydroxylation requires two soluble electron transfer proteins: rubredoxin (RubA) and rubredoxin reductase (RubB). RubB transfers electrons from NADH to RubA via its FAD1 cofactor, which subsequently delivers electrons to AlkB [21]. The corresponding genes *rubA* and *rubB* were annotated in the genome of *A. calcoaceticus* 21#. The canonical terminal oxidation pathway, first characterized in *Pseudomonas putida* GPo1, initiates with AlkB [22]. The degradation of n-alkanes occurs primarily through terminal oxidation and subterminal oxidation pathways under aerobic conditions, ultimately yielding H_2_O and CO_2_ [23]. Following alkane hydroxylation, the alcohol dehydrogenation phase generates corresponding aldehydes. Genomic analysis of *A. calcoaceticus* 21# revealed numerous alcohol dehydrogenase-encoding genes, including *ADH4*, *yiaY*, and *frmA*. Subsequent aldehyde dehydrogenation converts aliphatic aldehydes to fatty acids, facilitated by annotated genes such as *ald1* and *ALDH6A1*. The fatty acid-CoA ligase encoded by *fadD* then catalyzes the formation of acyl-CoA derivatives. β-oxidation proceeds via FadB and FadE, iteratively removing two-carbon units as acetyl-CoA and generating shortened acyl-CoA chains until complete oxidation to acetyl-CoA, which enters the tricarboxylic acid (TCA) cycle for terminal mineralization to H_2_O and CO_2_. The proposed mid-chain alkane degradation pathway in strain *A. calcoaceticus* 21# is depicted in Figure 1.

**Figure 2:**
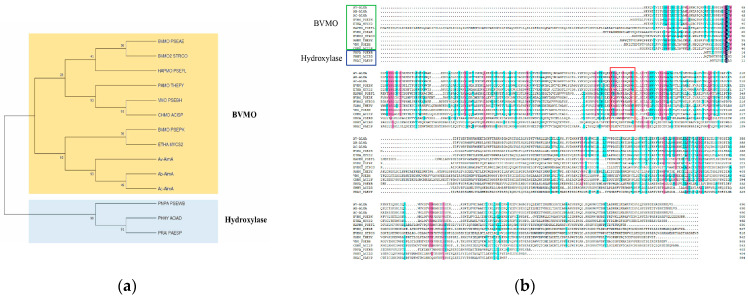
Phylogenetic and sequence analysis of AlmA homologs. (**a**) Phylogenetic evolutionary tree of AlmA and its homologous proteins. (**b**) Multiple sequence comparison of AlmA and its homologous proteins. Note: The red box highlights characteristic motifs confirming classification of the target protein within the Baeyer-Villiger monooxygenase family.

Long-Chain Alkane Degradation Genes. In current research, two representative genes for long-chain alkane degradation are *alma* [24] and *ladA* [25]. Genomic mining of *A calcoaceticus* 21# identified both *almA* and *ladA* homologs. To infer the physiological and biochemical roles of AlmA, a maximum likelihood (ML) phylogenetic tree was constructed using AlmA proteins from *A. calcoaceticus* 21# and *A. vivianii* KJ-1, along with nine reported BVMOs and three hydroxylases (Figure 2a). av-almA forms a distinct clade with Ac-AlmA and the functionally characterized BVMO from *A. baylyi* ADP1 (Ab-AlmA) [26], supported by a 93% bootstrap value (Figure 2a). This clade clusters definitively within the BVMO superfamily and exhibits unambiguous separation from hydroxylases (99% bootstrap support). The topology confirms that both av-almA and Ac-AlmA belong to the BVMO functional class, indicating AlmA functions as a BVMO rather than a hydroxylase. Multiple sequence alignment (MSA) further corroborated this inference, demonstrating conserved BVMO-specific motifs (FxxGxxxHxxxW(P/D) [27,28] in AlmA of *A. calcoaceticus* 21# (Figure 2b). Wang and Shao [29] demonstrated in vitro that an AlmA homolog from *Alcanivorax dieselolei* B-5 catalyzes terminal hydroxylation of alkanes, providing the first evidence in *Alcanivorax* that *almA* encodes an enzyme with alkane hydroxylase function. Conversely, a recent study by Yin et al. [26] revealed through physiological, biochemical, and bioinformatic analyses of AlmA that the enzyme from *A. baylyi* ADP1 participates in the subterminal oxidation pathway of alkane degradation, functioning as a BVMO. The high bootstrap support, sequence conservation, and intact catalytic motifs collectively demonstrate that av-almA operates as a BVMO in *A. vivianii* KJ-1.

Given these discrepant reports on AlmA functionality, the precise role of AlmA across bacterial genera remains unresolved. Considering our analyses, AlmA in *A. calcoaceticus* 21# and *A. vivianii* KJ-1 likely mediates subterminal oxidation of long-chain alkanes via BVMO activity. However, hydroxylases associated with subterminal oxidation are scarcely documented in *Acinetobacter* species, thus warranting further exploration of this enzymatic mechanism. Although *av-almA* and *ac-almA* exhibit high sequence similarity, functional divergence among homologous genes across bacterial strains necessitates empirical validation. This research specifically investigates its degradation genes for medium- to long-chain alkanes, conducting an in-depth exploration of their functional roles. These findings establish a theoretical foundation for the rational design of genetically engineered bacterial strains with enhanced alkane-degrading capabilities.

#### 3.1.2. Genes Associated with Aromatic Hydrocarbon Degradation

Aromatic hydrocarbons exhibit greater recalcitrance to biodegradation compared to alkanes. Under aerobic conditions, their degradation typically initiates with dioxygenase-mediated conversion to *cis*-dihydrodiol intermediates, followed by dehydrogenase-catalyzed oxidation to catechol derivatives. Subsequent aromatic ring cleavage by *ortho*- or *meta*-dioxygenases yields linear metabolites that enter the tricarboxylic acid (TCA) cycle [30].

**Figure 3:**
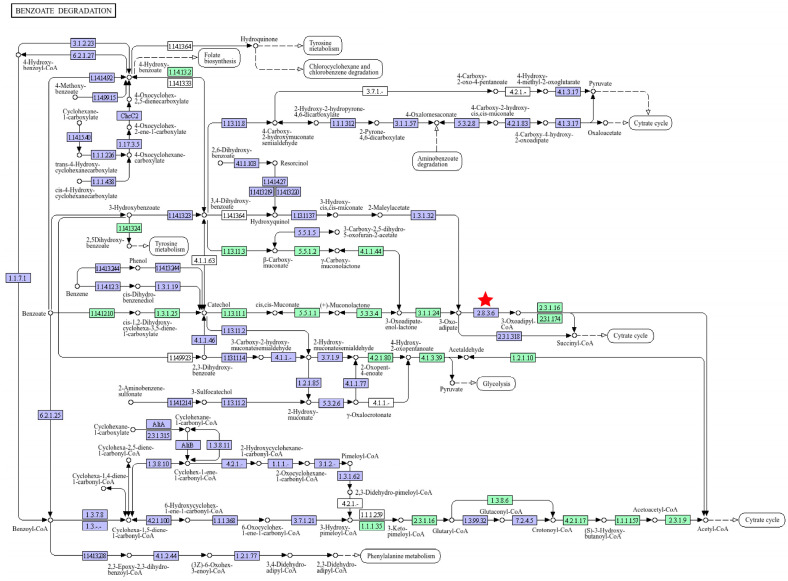
The metabolic pathway of parabens inferred in *A. calcoaceticus* 21#. The red star indicates that the gene at this location might be the *pcaJ* gene in 21#. The protein encoded by this gene has the function of connecting 3-Oxoadipate with coenzyme A. Green indicates the species-specific functional genes annotated in this organism.

Genes implicated in aromatic hydrocarbon degradation in *A. calcoaceticus* 21# are cataloged in Table 3. For instance, the *benABC* gene cluster encodes benzoate 1,2-dioxygenase, which converts benzoate to 2-hydro-1,2-dihydroxybenzoate (DHB). DHB is further oxidized to catechol by 1,2-dihydrodihydroxybenzoate dehydrogenase (encoded by *benD*) [31]. The *catA-catC* operon facilitates *ortho*-cleavage of catechol to β-ketoadipate enol-lactone [32]. β-Ketoadipate enol-lactone is hydrolyzed to β-ketoadipate by muconolactone hydrolase (encoded by *pcaD*) [33], which is subsequently metabolized to succinyl-CoA and acetyl-CoA via β-ketoadipate CoA-transferase (FadA) and β-ketoadipyl-CoA thiolase (PcaF), respectively, for TCA cycle entry [34]. During gene mining, catechol 1,2-dioxygenase, a key enzyme acting on the rate-limiting step of aromatic hydrocarbon degradation, was identified. This enzyme may contribute to the high Pyr degradation capacity observed in *A. calcoaceticus* 21#. KEGG pathway analysis revealed that these genes also mediate the degradation of monocyclic aromatic pollutants, including *p*-aminobenzoate, 2-fluorobenzoate, chlorobenzene, toluene, xylene, nitrotoluene, and ethylbenzene. The benzoate degradation pathway in *A. calcoaceticus* 21# is schematically summarized in Figure 3.

In addition to the aforementioned benzoate degradation genes, we identified additional aromatic hydrocarbon degradation genes in the genome of *A. calcoaceticus* 21#. Within the KEGG pathway for PAH degradation, the genes *pcaH* and *pcaG* were annotated, encoding the α and β subunits of protocatechuate 3,4-dioxygenase (EC 1.13.11.3), which catalyzes the conversion of protocatechuate to β-carboxymuconoyl-CoA. In the naphthalene degradation pathway, the genes *frmA* (encoding S-(hydroxymethyl) glutathione dehydrogenase, EC 1.1.1.284) and *yiaY* (encoding alcohol dehydrogenase, EC 1.1.1.1) were identified. Additionally, the genes *nagAb* and *ndoA* were annotated, encoding the ferredoxin component of the naphthalene dioxygenase multicomponent enzyme system (EC 1.14.12.12), which facilitates electron transfer during the initial dioxygenation of naphthalene. Systematic analysis of polycyclic aromatic hydrocarbon catabolic genes in this strain revealed functional gaps in the degradation pathway, particularly the absence of key proteins for meta-cleavage. This deficiency constrains its full bioremediation potential, thereby establishing a foundation for targeted genetic engineering to enhance contaminant mineralization capacity.

### 3.2. Hydrocarbon Degradation Capacities

**Figure 4:**
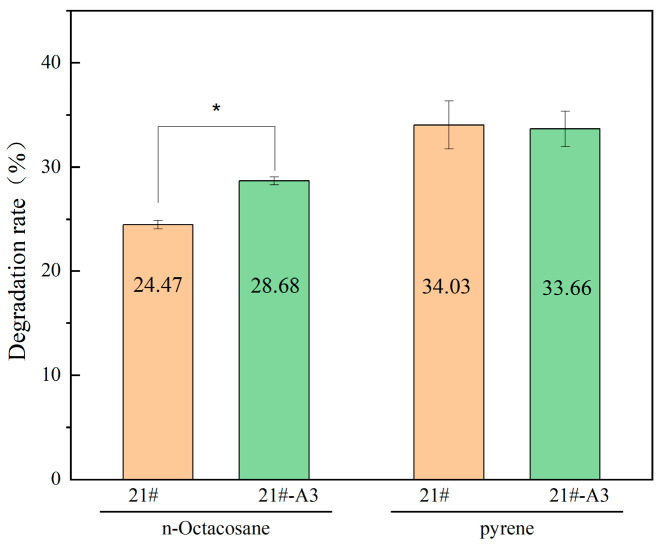
Hydrocarbon degradation efficiency of *A. calcoaceticus* strains. Note: Data represent mean ± SD. * *p* < 0.05 vs. 21# control group.

This study evaluated the hydrocarbon degradation capacity of *Acinetobacter calcoaceticus* and simultaneously investigated the impact of exogenous gene introduction on the degradation performance of the native strain. The engineered strain 21#-A3 showed a statistically significant increase in n-Octacosane degradation (28.68% vs. 24.47% in wild-type; *p* < 0.05), while Pyr degradation remained comparable (34.03% vs. 33.92%; not significant), which can be attributed to the integration of the *av-almA* gene from *A. vivianii* KJ-1, which up-regulated alkane catabolism. The pyrene degradation capacity remained statistically unchanged in the engineered strain 21#A3 compared to the parental strain. This demonstrates that integration of the exogenous long-chain alkane degradation gene *almA* neither significantly perturbed native catabolic gene expression nor enhanced aromatic metabolism, revealing functional compartmentalization between alkane and PAH degradation pathways (Figure 4).

Most crude oil-degrading bacteria exhibit a narrow substrate range. For instance, *Bacillus stearothermophilus* exclusively utilizes C_15_–C_17_ alkanes, while *A. calcoaceticus* Aca13 has been reported to degrade *n*-hexadecane and naphthalene [35]. In contrast, the recalcitrance of long-chain alkanes and toxicity of PAHs necessitate enhanced degradation strategies. While the metabolic mechanisms of known degradative bacteria for medium- and short-chain alkanes are well characterized, studies on ultra-long-chain alkanes remain limited [36]. We selected n-Octacosane and pyrene as representative substrates to investigate their degradation capabilities.

### 3.3. Construction of Prokaryotic Expression Vectors pET-28a(+)-av-almA-BH

**Figure 5:**
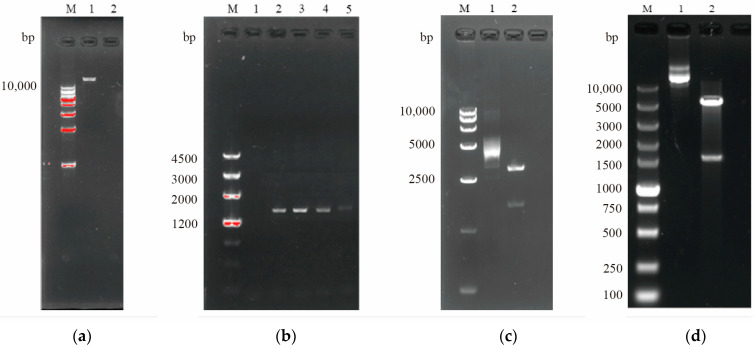
(**a**) Electropherogram of *A. vivianii* KJ-1 genomic DNA; (**b**) PCR products of *av-almA-BH*; (**c**) enzymatic verification; (**d**) double digestion identification of recombinant pET-28a(+)-*av-almA-BH* plasmid. Note: (**a**) M indicates the DNA marker, 1 indicates the extracted genomic DNA of KJ-1. (**b**) 1 indicates the negative control, where ddH_2_O was used as the template. 2–5 indicate the PCR amplification products at annealing temperatures of 58 °C, 60 °C, 62 °C, and 64 °C. (**c**) 1 indicates the pLB-*av-almA-BH* cloning vector, 2 indicates the restriction digestion results of pLB-*av-almA-BH*. (**d**) M indicates the DNA marker, 1 indicates the pET28a(+)-*av-almA-BH* plasmid carrier, and 2 indicates the result of enzyme digestion.

Using genomic DNA of *A. vivianii* KJ-1(Figure 5a) as the template, the optimal annealing temperature for primers F-B-*av-almA-BH* and R-H-*av-almA-BH* was determined as 60 °C through temperature gradient PCR (Figure 5b), successfully amplifying the *av-almA-BH* with the termination codon TAA removed. After screening on ampicillin-containing plates, plasmid extraction and electrophoresis identified a recombinant plasmid pLB-*av-almA-BH* of approximately 4500 bp (Figure S3). Restriction enzyme digestion of the plasmid yielded two distinct fragments (2800 bp corresponding to the pLB cloning vector and 1500 bp corresponding to the *av-almA-BH*, Figure 5c), confirming the successful construction of the recombinant cloning vector. The minor discrepancy resulted from the intentional deletion of the termination codon at the 3′-end of *almA* to enable the expressed protein to carry a C-terminal 6×His tag encoded by the pET28a(+) vector. This modification preserved protein functionality while facilitating subsequent purification.

The recombinant cloning vector pLB-*av-almA-BH* cloning plasmid (Figure S3) was extracted from *E. coli* BL21(DE3)-*av-almA-BH* in order to clone the *av-almA* gene and construct the expression vector pET28a(+)-*av-almA-BH*; the result showed that *av-almA* was successfully inserted into the pET28a(+) vector (Figure 5d). The sequence of the cloned *av-almA* gene had 85.48% similarity with the *almA* gene (EF212873) from *Acinetobacter* sp. DSM 17874 [24] and 53.96% similarity with that from *Alcanivorax dieselolei* B-5 (FJ263134) [29]. These two genes were both shown to be involved in the degradation of long-chain alkanes.

### 3.4. Functional Identification of the Recombinant AlmA Proteins

**Figure 6:**
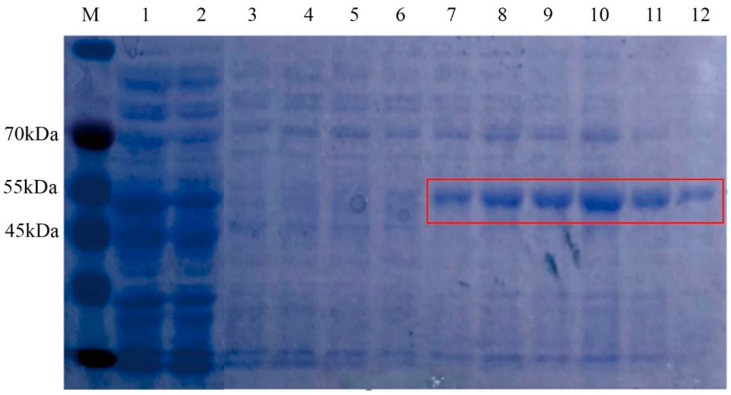
SDS-PAGE plot of purified recombinant AlmA protein. Note: M indicates protein molecular weight standard; 1, total protein; 2, permeate; 3–6, different batches of wash buffer; 7–12, different batches of elution buffer.

*E. coli* BL21(DE3)-*av-almA-BH* showed a distinct band near 55 kDa after 4 h of induction at 37 °C with 0.1 mM IPTG. This band was equal in size to the protein molecular weight prediction of 55.8 kDa, which was determined to be the target protein band (Figure S4). Subsequently, the affinity chromatography on a nickel column was used to purify the AlmA protein. The purification results are shown in Figure 6; lanes 7 to 12 are the purified proteins. Purified enzyme solutions were employed for enzyme activity determination. The purified enzyme condition can avoid the influence of coenzymes and non-target proteins contained in the protein solution. Therefore, the measured enzyme activity is more accurate, which gave a better understanding of the properties of the target proteins.

**Figure 7:**
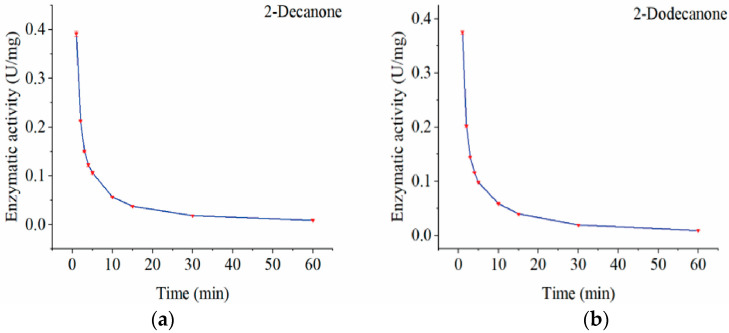
Enzymatic activity of AlmA on 2-decanone (**a**) and 2-dodecanone (**b**) at different times.

Figure 7 shows that the recombinant AlmA protein showed high Baeyer–Villiger monooxygenase (BVMO) activity by reaching 0.39 (U/mg) for 2-decanone and 0.37 (U/mg) for 2-dodecanone within 1 min. The enzyme activity remained at 0.11 U/mg and 0.098 U/mg after 5 min but significantly decreased within 10 min. This rapid decline might be due to the swift binding of NADPH to the enzyme’s binding site during the initial reaction period, allowing AlmA to be fully utilized and producing NADP+. After 10 min, the enzyme activity became very low and nearly constant, possibly because NADP+ did not detach easily from the enzyme’s binding site. Since NADP+ is a competitive inhibitor of NADPH, it limited the enzyme activity [37,38]. The transient catalytic activity of AlmA is primarily attributed to NADP^+^-mediated allosteric inhibition and rapid cofactor depletion, which critically constrain scalability. Addressing this limitation requires integrated enzyme engineering strategies—such as rational design of cofactor-binding domains and construction of NADPH-regenerating fusion enzymes—that maintain catalytic efficiency while enabling scalable alkane functionalization.

**Figure 8:**
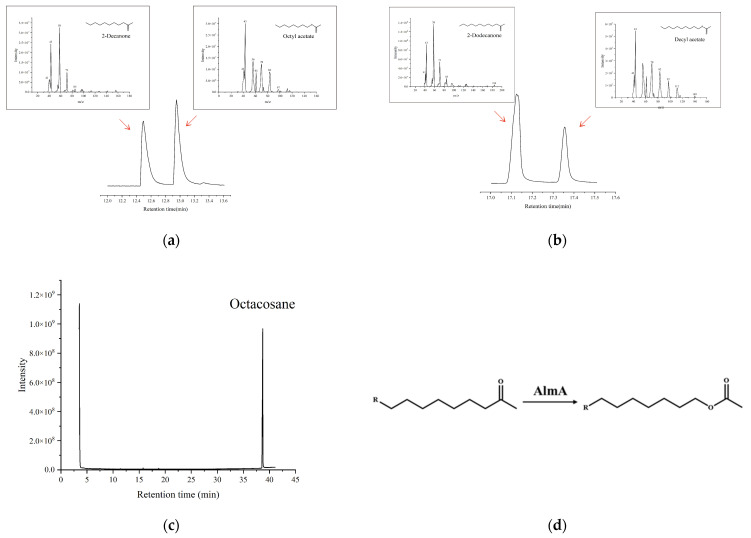
GC-MS identification of metabolites of the enzymatic reaction of AlmA protein. (**a**) Chromatograms and mass spectra of 2-decanone and octyl acetate; (**b**) chromatograms and mass spectra of 2-dodecanone and decyl acetate; (**c**) chromatograms of n-Octacosane; (**d**) Baeyer–Villiger reaction of aliphatic ketones catalyzed by AlmA.

The results of metabolite analysis are shown in Figure 8. Firstly, Figure 8a shows the conversion of 2-decanone to octyl acetate and Figure 8b shows the conversion of 2-dodecanone to decyl acetate. In contrast, Figure 8c indicates that only n-Octacosanoids were detected, with no corresponding alcohol identified. The results demonstrate that the AlmA protein can convert aliphatic ketones to the corresponding esters, suggesting involvement in the subterminal oxidation pathway of alkane degradation, but does not have the ability to hydroxylate alkanes.

In vitro studies revealed that AlmA homologs from *Alcanivorax diesolei* B-5 could catalyze the terminal hydroxylation of C_10_~C_36_ alkanes and participate in the terminal oxidation pathway of alkane degradation [29]. Additionally, Yin et al. [26] demonstrated that AlmA proteins from *A. baylyi* ADP1 could convert aliphatic ketones to their corresponding esters, exhibiting BVMO activity. Minerdi et al. [39] showed that AlmA proteins derived from *Acinetobacter radioresistens* S13, an AlmA protein homolog, could catalyze the Baeyer–Villiger oxidation of 4-phenyl-2-butanone (a non-alkane metabolite). GC-MS analysis of metabolites associated with the AlmA protein identified octyl acetate and decyl acetate-metabolic products derived from 2-decanone and 2-dodecanone, respectively-while no metabolites of n-Octacosane were detected. Quantitative degradation assays confirmed no significant reduction in n-Octacosane levels, consistent with the absence of derivatives in GC-MS profiling. These results provide further evidence that AlmA functions as a BVMO in alkane degradation. Moreover, the data suggests that Acinetobacter species likely degrade long-chain alkanes via the subterminal oxidation pathway rather than the terminal oxidation pathway, as subterminal oxidation demonstrates superior carbon acquisition efficiency during the conversion of long-chain alkanes to medium/short-chain alcohols. The results of this study indicate that the AlmA protein possesses BVMO activity, demonstrating that while *almA* genes from different genera may share sequence similarities, their functions can vary. In subsequent experiments, we will perform quantitative expression profiling to compare endogenous *almA* of *A*. *calcoaceticus* 21# with heterologously expressed *av-almA*.

## 4. Conclusions

In this study, integrated analysis of whole-genome sequencing and metabolic pathways enabled genomic mining of petroleum hydrocarbon degradation genes in strain 21#, identifying key genes including *alkB-1* and *alkB-2* for medium-chain alkane degradation, *almA* and *ladA* for long-chain alkane degradation, and catechol 1,2-dioxygenase acting as the rate-limiting enzyme in aromatic degradation. Phylogenetic evidence suggests AlmA in *A. calcoaceticus* 21# and *A. vivianii* KJ-1 likely mediates subterminal oxidation of long-chain alkanes via BVMO activity. To assess the impacts of exogenous gene integration on native hydrocarbon degradation capacity, degradation efficiencies of wild-type *A. calcoaceticus* 21# and the engineered strain 21#-A3 were quantified: wild-type 21# exhibited high degradation of both n-Octacosane and pyrene, while 21#-A3 showed significantly enhanced n-Octacosane degradation. Critically, the standalone AlmA enzyme remains nonfunctional due to incomplete catalytic cascade; only when integrated into strain 21#’s native metabolic framework does increased expression drive efficient substrate conversion. Leveraging this enhancement, the expression vector pET-28a(+)-*av-almA-BH* was constructed for functional validation. Successful expression and purification of AlmA from *A. vivianii* KJ-1 revealed no activity toward long-chain alkanes but high enzymatic activity toward 2-decanone (0.39 U/mg) and 2-dodecanone (0.37 U/mg). GC-MS metabolite analysis detected octyl acetate (from 2-decanone) and decyl acetate (from 2-dodecanone), with no n-Octacosane derivatives, reconfirming AlmA’s BVMO function in alkane degradation. Alkane degradation relies on rate-limiting hydroxylation, while aerobic aromatic degradation is constrained by catechol 2,3-dioxygenase for meta-cleavage. Although *almA* integration enhanced long-chain alkane degradation in 21#-A3, KEGG analysis revealed gaps in polycyclic aromatic hydrocarbon pathways. Future work should integrate *C23O* or construct microbial consortia to expand the bioremediation potential for complex aromatic pollutants.

## Figures and Tables

**Table 1:** Bacterial strains, plasmids and primers.

Strains or Plasmids	Characteristics	Source
*A. calcoaceticus* 21#	Wild-type, with petroleum degradation capability (CGMCC NO.31080) (GenBank accession: CP196955)	Lab store
*A. calcoaceticus* 21#-A3	Genetically engineered bacterium, with the *almA* gene from KJ-1	Lab store
*A. vivianii* KJ-1	Wild-type, with diesel degradation capability (GenBank accession: CP085083)	Lab store
*E. coli* DH5α	Suitable for efficient gene cloning, ensure stable inheritance of high copy plasmids	Vazyme-China
pLB-vector	Ampr, containing lethal gene at MCS	TianGen-China
pLB-*av-almA-BH*	Ampr, pLB vector containing gene-*av-almA-BH*	This study
*E. coli* DH5α-*av-almA-BH*	Ampr, containing vector pLB-*av-almA-BH*	This study
pET-28a(+)	Kanr, containing T7 promoter and 6×His Tag	Lab store
pET-28a(+)-*av-almA-BH*	Kanr, pET-28a(+) vector containing gene-*av-almA-BH*	This study
*E. coli* BL21(DE3)-*av-almA-BH*	Kanr, containing vector pET-28a(+)-*av-almA-BH*	This study

**Table 2:** Putative genes related to medium chain alkane degradation in *A. calcoaceticus* 21#.

KEGG ID	Gene Name	Annotation
K00496	*alkB1_1*	Alkane 1-monooxygenase
K00496	*alkB1_2*	Alkane 1-monooxygenase
—	*rubA*	Rubredoxin
—	*rubB*	Rubredoxin reductase RubB
K13980	*ADH4*	Alcohol dehydrogenase
K13954	*yiaY*	Alcohol dehydrogenase
K00121	*frmA*	S-(hydroxymethyl) glutathione dehydrogenase/alcohol dehydrogenase
K00138	*ald1*	Long-chain-aldehyde dehydrogenase
K00140	*ALDH6A1*	Methylmalonate-semialdehyde dehydrogenase
K01897	*fadD*	Long-chain-fatty-acid-CoA ligase
K00249	*AFT10-1*	Acyl-CoA dehydrogenase
K00255	*ACADL*	Acyl-Coa dehydrogenase
K06445	*fade*	Acyl-coenzyme A dehydrogenase
K01825	*fadB*	Fatty acid oxidation complex subunit alpha

**Table 3:** Putative genes related to aromatic hydrocarbon degradation in *A. calcoaceticus* 21#.

KEGG ID	Gene Name	Annotation
K03381	*catA*	Catechol 1,2-dioxygenase
K03464	*catC*	Muconolactone D-isomerase
K01856	*catB*	Muconate cycloisomerase
K13954	*yiaY*	Alcohol dehydrogenase
K05549	*benA*	Benzoate/toluate 1,2-dioxygenase subunit alpha
K05550	*benB*	Benzoate/toluate 1,2-dioxygenase subunit beta
K05784	*benC*	Benzoate/toluate 1,2-dioxygenase reductase component
K05783	*benD*	Dihydroxycyclohexadiene carboxylate dehydrogenase
K01055	*pcaD*	3-oxoadipate enol-lactonase
K07823	*pcaF*	3-oxoadipyl-CoA thiolase
K00449	*pcaH*	Protocatechuate 3,4-dioxygenase, beta subunit
K0048	*pcaG*	Protocatechuate 3,4-dioxygenase, alpha subunit
K00121	*frmA*	S-(hydroxymethyl)glutathione dehydrogenase/alcohol dehydrogenase
-	*nagAb*	Naphthalene 1,2-dioxygenase/salicylate 5-hydroxylase systems
K05710	*ndoA*	Naphthalene 1,2-dioxygenase system, ferredoxin component
-	*tcpC*	6-chlorohydroxyquinol 1,2-dioxygenase

## Data Availability

The original contributions presented in this study are included in the article/Supplementary Materials. Further inquiries can be directed to the corresponding authors.

## Supplementary Materials

The following supporting information can be downloaded at: https://www.mdpi.com/article/10.3390/microorganisms13081953/s1, Figure S1. Distribution of gene length of strain *A. calcoaceticus* 21#. Figure S2. KEGG annotation of strain *A. calcoaceticus* 21#. Figure S3. Electrophoretic profile of recombinant cloning vector pLB-*av-almA-BH.* Figure S4. Electrophoretic profile of recombinant protein AlmA.
