# Supplementary material for: Insights into the Genomic Architecture and Improvement of the Capabilities of Acinetobacter calcoaceticus for the Biodegradation of Petroleum Hydrocarbons
Source: Microorganisms. 2025 Aug 21;13(8):1953. doi: 10.3390/microorganisms13081953 (PMC12388405; doi:10.3390/microorganisms13081953)
Supplement: Supplementary file 1 [file microorganisms-13-01953-s001.zip › microorganisms-3771719-supplementary.pdf]

Supplementary Materials

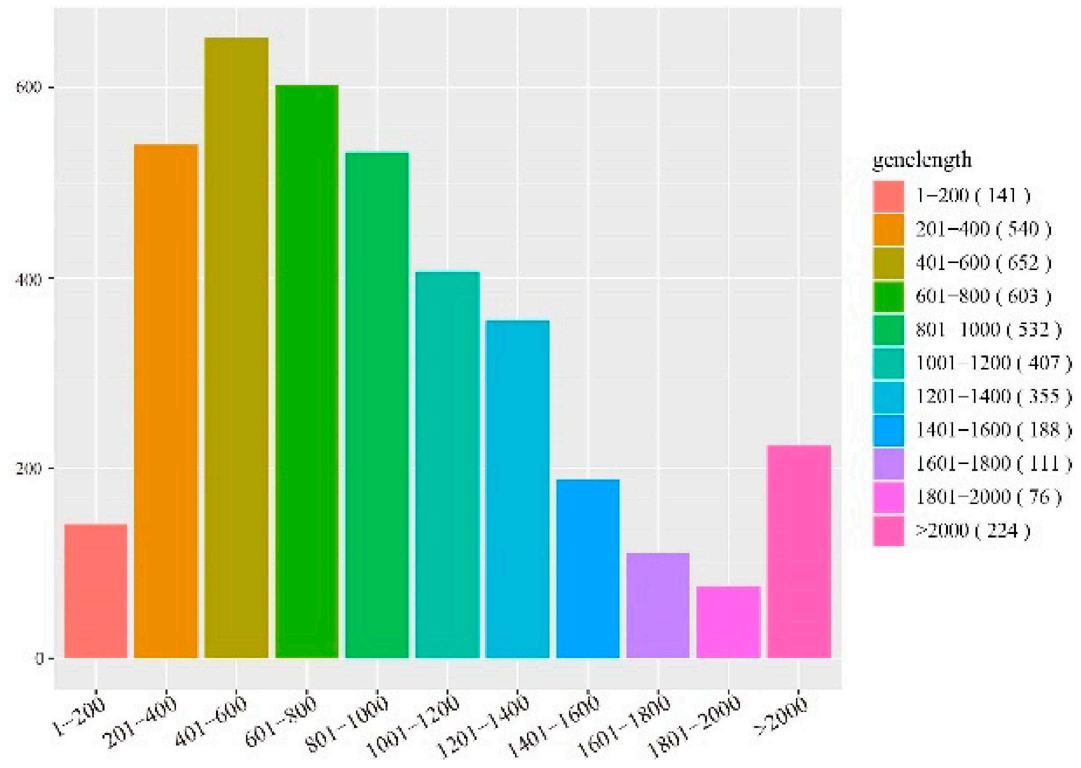

Figure S1. Distribution of gene length of strain *A. calcoaceticus* 21#.

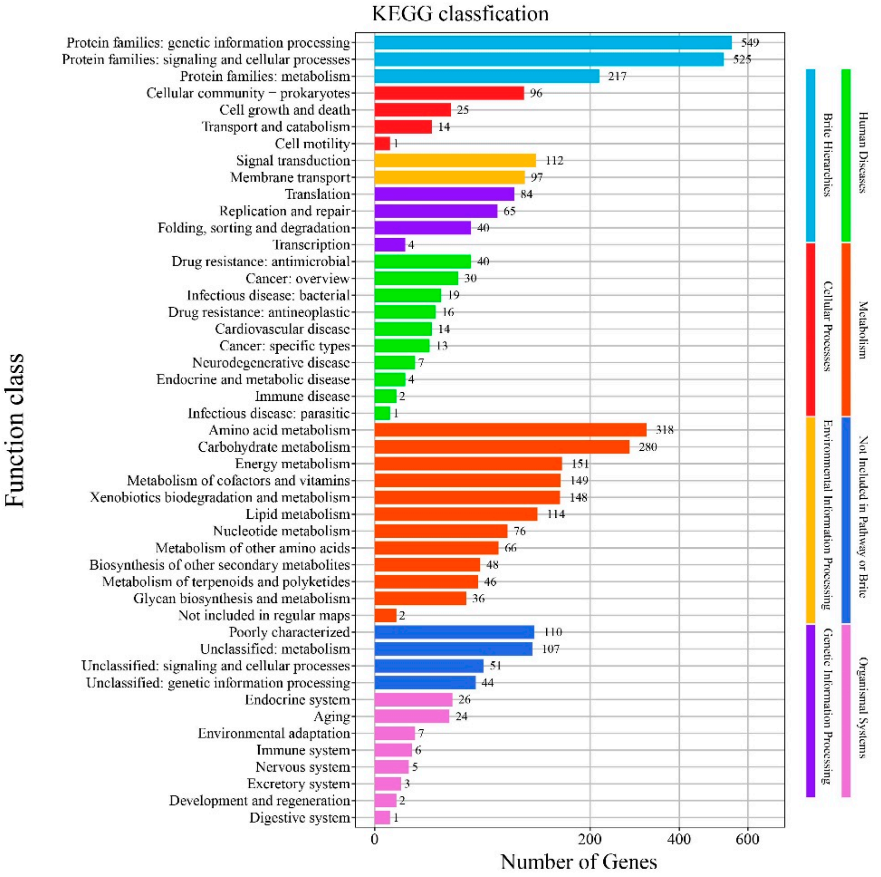

Figure S2. KEGG annotation of strain *A. calcoaceticus* 21#.

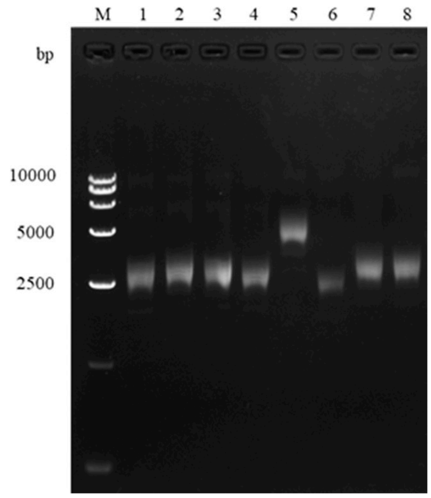

Figure S3. Electrophoretic profile of recombinant cloning vector pLB-*av*-*almA*-*BH*.
Note: M indicates DNA marker； 1-8 indicates the extracted pLB-*av*-*almA*-*BH* cloning Vector.

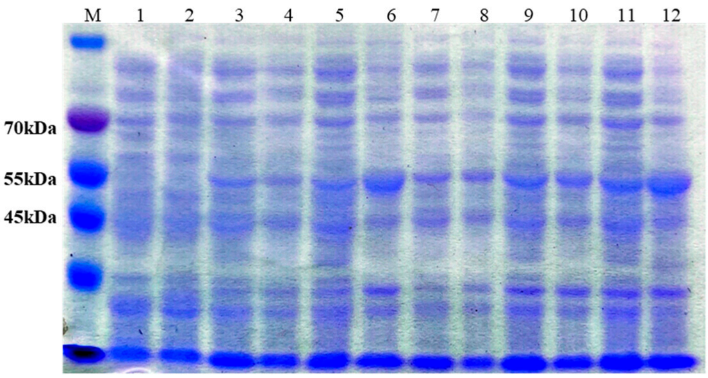

Figure S4. Electrophoretic profile of recombinant protein AlmA.
Note: M: protein molecular weight standard. 1-2 are soluble and insoluble expression results after *E.coli* BL21(DE3)-pET28a(+) induction, respectively. 3, 5, 7, 9, and 11 are soluble expression under *E. coli* BL21(DE3)-*av-almA-BH* uninduced, 20°C, 0.2 mM IPTG; 20°C, 0.1 mM IPTG; 16°C, 0.1 mM IPTG and 37°C, 0.1 mM IPTG induced conditions, respectively. 4, 6, 8, 10, and 12 are insoluble expression under *E. coli* BL21(DE3)-*av-almA-BH* uninduced, 20°C, 0.2 mM IPTG; 20°C, 0.1 mM IPTG; 16°C, 0.1 mM IPTG and 37°C, 0.1 mM IPTG induced conditions, respectively.
